# Cell Survival, Death, and Proliferation in Senescent and Cancer Cells: the Role of (Poly)phenols

**DOI:** 10.1016/j.advnut.2023.05.014

**Published:** 2023-06-02

**Authors:** Vincenzo Sorrenti, Alessandro Buriani, Stefano Fortinguerra, Sergio Davinelli, Giovanni Scapagnini, Aedin Cassidy, Immaculata De Vivo

**Affiliations:** 1 Department of Pharmaceutical and Pharmacological Sciences, University of Padua, Padua, Italy; 2 Maria Paola Belloni Center for Personalized Medicine, Padova, Italy; 3 IRCCS SDN, Napoli, Italy; 4 Department of Medicine and Health Sciences “V. Tiberio,” University of Molise, Campobasso, Italy; 5 Institute for Global Food Security, Queen’s University Belfast, Belfast, Northern Ireland; 6 Department of Epidemiology, Harvard T. H. Chan School of Public Health, Boston, MA, United States

**Keywords:** polyphenols, telomere, telomerase, antiaging, senolytics, anticancer, longevity

## Abstract

Cellular senescence has long been considered a permanent state of cell cycle arrest occurring in proliferating cells subject to different stressors, used as a cellular defense mechanism from acquiring potentially harmful genetic faults. However, recent studies highlight that senescent cells might also alter the local tissue environment and concur to chronic inflammation and cancer risk by secreting inflammatory and matrix remodeling factors, acquiring a senescence-associated secretory phenotype (SASP). Indeed, during aging and age-related diseases, senescent cells amass in mammalian tissues, likely contributing to the inevitable loss of tissue function as we age. Cellular senescence has thus become one potential target to tackle age-associated diseases as well as cancer development. One important aspect characterizing senescent cells is their telomere length. Telomeres shorten as a consequence of multiple cellular replications, gradually leading to permanent cell cycle arrest, known as replicative senescence. Interestingly, in the large majority of cancer cells, a senescence escape strategy is used and telomere length is maintained by telomerase, thus favoring cancer initiation and tumor survival. There is growing evidence showing how (poly)phenols can impact telomere maintenance through different molecular mechanisms depending on dose and cell phenotypes. Although normally, (poly)phenols maintain telomere length and support telomerase activity, in cancer cells this activity is negatively modulated, thus accelerating telomere attrition and promoting cancer cell death. Some (poly)phenols have also been shown to exert senolytic activity, thus suggesting both antiaging (directly eliminating senescent cells) and anticancer (indirectly, via SASP inhibition) potentials. In this review, we analyze selective (poly)phenol mechanisms in senescent and cancer cells to discriminate between in vitro and in vivo evidence and human applications considering (poly)phenol bioavailability, the influence of the gut microbiota, and their dose-response effects.

**Statement of Significance**

To our knowledge, this review for the first time addresses 2 apparently contradicting effects of nutritional (poly)phenols on cancer and senescent cells, where there is evidence of both inhibition and promotion of proliferation and cell death. The review pieces all the information together to propose the harmonization of the 2 phenomena.

## Introduction

In 1961, Leonard Hayflick identified the human cell’s capability to divide and repair to approximately between 40 and 60 times in cell culture before reaching senescence, thus the “Hayflick limit” [1]. Cell division is the most complex and energetically exploiting phenomenon in a cell’s cycle, stressing its genetic components and exposing the cell to genetic errors with time. Cellular senescence can be defined as a permanent state of cell cycle arrest that occurs in proliferating cells subject to different stresses, including overreplication. Senescence can be considered a cellular defense mechanism against the acquisition of potentially harmful transcriptional errors. An enhanced secretory phenotype, failure to re-enter the cell cycle in response to mitogenic stimuli, and resistance to cell death characterize the senescent state, which can take place in several tissues during injury, tissue remodeling, cancer, and aging [2]. Cellular senescence may be derived from an extrinsic pathway that is telomere-independent known as stress-induced premature senescence (SIPS) or an intrinsic one that is telomere-dependent and is known as replicative senescence [3]. although the latter is regulated via a biological clock linked to the progressive shortening of telomere repetitive DNA sequences (tandem TTAGGG repeats), SIPS is not programmed but is related to cellular stress.

Although senescence represents a physiological strategy to suppress tumorigenesis, recent studies demonstrate how senescent cells can secrete several inflammatory cytokines, growth factors, chemokines, and matrix remodeling factors, acquiring a senescence-associated secretory phenotype (SASP). SASP alters the local tissue environment, contributing to chronic inflammation and possibly cancer-permissive or even cancer-promoting environments [4]. Apart from beneficial effects in a few situations, SASP is generally detrimental during aging, accelerates age-related conditions, and increases therapeutic resistance and inefficacy of cancer treatments [5,6]. In certain rare cases, cells have been shown to even develop oncogenic changes, bypass senescence, and maintain stable but usually shortened telomere lengths by exploiting telomere maintenance and telomere protection mechanisms, finally developing genomic instability and continuing to divide toward the development of the malignant phenotype [7].

In general, anticancer treatments frequently induce an off-target effect in the tumor microenvironment with a dramatic impact on disease progression known as therapy-induced senescence (TIS) of cells. By indirectly fueling the repopulation of remaining cancer cells and bestowing cancer resistance, TIS can induce posttreatment tumor relapse and metastasis substantially driven by the SASP [6]. Targeting treatments to remove senescent cells efficiently and selectively before they provide such pernicious effects is becoming an effective solution. One of the most promising approaches is senotherapy. Senolytic compounds are a group of natural and synthetic drugs developed to directly remove senescent cells, providing efficacy in cancer cotreatments by interfering with cancer survival-associated pathways, inducing a selective removal of senescent cells, known as senescent cell antiapoptotic pathways [8].

Phytocomplexes can provide compounds able to modulate multiple cellular functions both directly through anti-inflammatory and redox-modulating activities and indirectly by interacting with key epigenetic mechanisms on gene transcription of proteins involved in mechanisms related to survival pathways and cell replication [9]. In particular, (poly)phenols, represent an abundant heterogeneous class of compounds present in phytocomplexes of vegetables, fruits, tea, chocolate, and cereals, among others.

Once ingested, (poly)phenol bioavailability differs considerably from one to another, depending on the degree of polymerization, their chemical structure, and the sugar moiety, and the (poly)phenols most present in the diet are not always those that allow the highest concentrations of active metabolites to be achieved in target tissues [10]. In fact, (poly)phenol bioavailability varies greatly not only among (poly)phenols but for some of them also depending on the forms present in food. The estimation of the individual exposure to the (poly)phenol metabolome is complex and needs to be further improved with advanced techniques [11]. The plasma concentrations of total metabolites range from 0 to 4 μmol/L with an intake of 50 mg aglycone equivalents. A relevant article by Manach et al. [10] highlighted that gallic acid and isoflavones are the most well-absorbed (poly)phenols, followed by catechins, flavanones, and quercetin glucosides. Proanthocyanidins, anthocyanins, and the galloylated tea catechins are the least well-absorbed (poly)phenols. Once in the colon, some (poly)phenols undertake microbial fermentation producing small (poly)phenol bioactive metabolites, which, in part, might be responsible for their health benefits [12].

(Poly)phenols’ metabolic fate is thus affected by different factors: interindividual variations in (poly)phenol metabolism caused by genetic polymorphisms of transporters, metabolic enzymes, efflux pumps, etc.; bidirectional interaction with the gut microbiota, which seems to be the primary driver of this interindividual variation; and antagonism or synergies with dietary nutrients or other xenobiotics, among others [[12], [13], [14]]. Several studies have shown that (poly)phenols such as quercetin, curcumin, resveratrol, and epigallocatechin-3-gallate (ECGC), among others, can act as senolytics and interfere with aging processes by promoting DNA repair, selectively removing senescent cells, and inhibiting oxidative stress, inflammatory processes, and telomere shortening [15,16]. Interestingly, different from what happens in normal cells, in cancer cells, (poly)phenols exhibit inhibitory mechanisms on telomerase activity and cell survival pathways, potentially inducing cancer cell death. The mechanisms by which (poly)phenols lead to these paradoxical effects are not fully understood and cannot be explained without considering the pleiotropic nature of these phytochemicals that act on many different molecular targets in several different ways that reinforce or mitigate specific cellular functions [9]. The final result of these complex interactions is also dependent on the larger biological context, where activation or suppression of the same molecular machinery can be functionally associated with different biological scopes. The same molecular perturbation of a given pathway can thus lead to different results when it is differently related with the functional machinery of the cell [17]. Regardless of the effect on telomerase, in the case of senolytic (poly)phenols, the elimination of senescent cells could represent the one therapeutic target for both aging-associated diseases, directly acting on the aging tissues, and indirectly on cancer cells, acting on cancer-promoting SASP. In this review, studies analyzing (poly)phenol selective mechanisms based on the cellular phenotype will be discussed, and explanations proposed.

## Potential (Poly)phenol Targets in Senescent and Cancer Cells

### Telomeres

**FIGURE 1:**
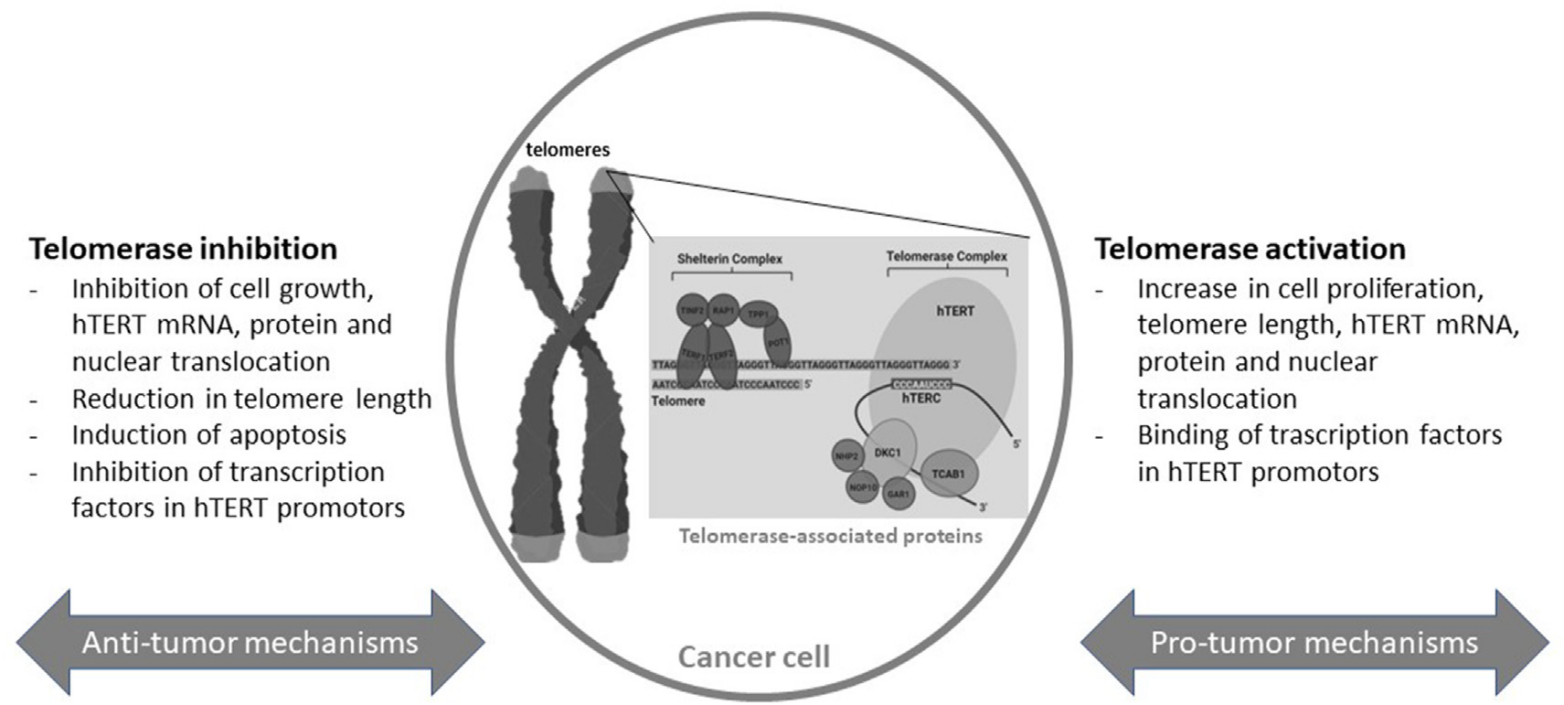
Telomerase-related mechanisms of cancer growth and inhibition. hTERT, human telomerase reverse transcriptase.

Telomeres are dynamic nucleoprotein structures containing repeated nucleotide sequences in terminal regions (tandem TTAGGG repeats) of linear chromosomes associated with several proteins, which form a complex known collectively as the “Shelterin,” that maintain the genomic integrity of a cell [18] by shielding and protecting the terminal end of linear chromosomes (Figure 1) [19]. Due to the incapacity of DNA polymerase to completely synthesize the daughter strand at chromosomal ends, telomeric DNA is lost with each cell division [20]. On average, human telomeres lose 50 to 100 base pairs per mitotic division, thereby reducing the cell’s ability to replicate [21,22].

Telomere attrition, the base loss from telomeres, is mainly mediated by reactive oxygen species (ROS) released during inflammation, which induces chemical alterations to pyrimidines and purines. Once a critical telomere length is reached, the cell undergoes a proliferation block where it stops dividing (replicative senescence) or activates programmed cell death [23,24]. Telomeres protect chromosome ends, but when terminal nucleotide sequences become critically short following progressive cell divisions, they lose their protective function and DNA damage responses can be elicited, thus leading to cellular senescence. The telomere theory of cellular aging and senescence indicates how cells implement mechanisms to estimate the number of divisions and detect when the end of replication is necessary. In age-related diseases, tissue accumulation of senescent cells could lead to the loss of tissue function, as seen with aging. In some cases, cells can gain oncogenic transformation and bypass senescence, maintaining stable but usually shortened telomere lengths. These cells can indeed exploit telomere maintenance and telomere protection mechanisms, develop genomic instability, and continue to divide, eventually developing a malignant phenotype [7,[25], [26], [27]]. Telomeres are crucial for cancer cell survival, and the vast majority of tumors are maintained by the enzyme telomerase [28,29].

Telomere length expression and maintenance are regulated at the transcriptional, posttranscriptional, and epigenetic levels through molecular machinery mostly associated with telomerase function, an enzymatic complex whose RNA component acts as the template for repeat addition operated by the enzyme telomerase reverse transcriptase (TERT) [30].

Telomerase is a ribonucleoprotein enzyme that extends telomeres by adding telomere repeats and is constitutively expressed to allow long-term cell division and viability. Telomerase activity decreases during the cellular lifespan and normally becomes silent in adult cells [31]. Malignant cells can achieve proliferative immortality by reactivating telomerase, via upregulation of the human TERT gene (*hTERT*). hTERT, together with the noncoding human telomerase RNA, a template for the addition of telomeric repeats to chromosome ends, is part of the telomerase ribonucleoprotein complex [32,33]. Although the vast majority of cancer cells earn immortality by reactivating telomerase to stabilize their shortened telomere lengths [34,35], in ∼10% of tumors, the pathway eliciting the alternative lengthening of telomeres enables cancer cells to reverse telomere attrition and bypass senescence using telomeric templates for synthesis and forming DNA repair hot spots [27]. Targeting telomerase in selective senescent cancer cells without altering telomerase in normal cells seems to be a promising new strategy for anticancer therapy [36,37].

Given its central role in telomerase activity, hTERT regulation has been extensively investigated. hTERT inhibition via telomerase inhibition can be an effective strategy to progressively achieve telomere shortening and, eventually, cancer cell death.

Several strategies of telomerase inhibition targeting hTERT have been developed, including antisense oligonucleotides small molecule inhibitors, gene therapies, immunotherapies, and regulatory mechanisms of alternative RNA splicing, some with strong therapeutic potential [35,38]. A thorough characterization of these mechanisms may allow a better comprehension of the telomere length regulation, thus opening the way to identify novel biomarkers for early detection of disease, prognosis, and new pharmacological targets for the development of therapeutics [7,25,28,39,40].

The rate of telomere loss is affected by factors other than mitotic replication, and telomere length is damaged by oxidative stress and inflammation [41]. ROS released during inflammation or chemical alterations to nucleic bases caused by oxidative stress may induce telomere damage [42]. Conditions that can contribute to oxidative stress, like chronic stress, obesity, and smoking, have also been related to shorter telomeres [43]*.* On the other hand, a healthy diet with plant-based foods has been associated with a more stable telomere length [44], playing a role in telomere maintenance [45,46]. A healthy diet has also been suggested to attenuate telomere shortening and delay the onset of age-related disorders and longevity [47]. A diet rich in fiber and high in fruit and vegetables has also been associated with longer telomere length, given their anti-inflammatory effects. In contrast, a diet high in processed foods and *trans* fatty acids, which are associated with inflammation and oxidative stress, accelerates telomere shortening, cellular aging, and cancer progression [48,49]. Recently, different lifestyle factors that can potentially protect telomeres have been highlighted [49]. People who follow a healthy lifestyle by adhering to the Mediterranean diet [50], increasing their physical activity [51], and practicing meditation [52] have been shown to have longer telomeres than those who do not. In particular, the Mediterranean diet is rich in (poly)phenols, which have been associated with longer telomeres and reduced risk of age-related disorders [15,[53], [54], [55], [56]]. In vitro and in vivo studies have shown curcumin, quercetin, and resveratrol, among others, may maintain telomere length by modulating inflammation and oxidative stress [15,53].

### SASP

Recent evidence has demonstrated how senescent cells alter the local tissue environment, favoring chronic inflammatory processes and cancer development, by secreting a plethora of inflammatory cytokines, growth factors, chemokines, and matrix remodeling factors [4]. This secretory phenotype of senescent cells, known as SASP, has been observed both in cultured cells and in vivo [57]. By acting in a paracrine fashion, SASP factors strengthen the senescence of senescent cells themselves and induce the senescence of surrounding cells (paracrine senescence) [58]. However, chemokines and cytokines released from senescent cells can act on immune cells, such as natural killer cells, and macrophages, leading to the elimination of senescent cells [59]. Recently, senescent cells have been shown to transiently appear during organ development in mammals, where SASP factors participate in the differentiation of surrounding cells and the removal of unnecessary cells [60]. Nonetheless, “chronic” SASP has been demonstrated to induce senescence in adjacent young cells, contributing to tissue dysfunction and paradoxically favoring tumorigenesis [4]. These effects are tumor stage-dependent. In precancerous cells (or in a very early stage of cancer), which are in a senescent state, SASP seems to be predominantly tumor suppressive, recruiting immune cells to remove precancerous senescent cells (known as “senescence surveillance”). However, in advanced cancer, SASP factors favor cancer cell proliferation, promoting tumor progression [61]. In general, (poly)phenols may act upstream and downstream to inhibit either SASP factors or their effects (62). Quercetin, fisetin, naringenin, hesperetin, hesperidin, kaempferol, rutin, apigenin, luteolin, EGCG, and procyanidin C1, among others, have shown antisenescence effects in different in vitro and in vivo studies [63].

### p53/p21cip1 and p16INK4A/Retinoblastoma (Rb)

In stress-induced senescence, the severity and duration of the stressful stimulus appear to play a key role; in fact, it seems that senescence requires a stable stimulus, whereas transient stimuli are associated only with a transient arrest of growth, allowing the cell to repair the damage. More severe stimuli instead induce apoptosis. The p16INK4A/Rb and/or p53/p21cip1 tumor suppressor pathways are the most extensively studied pathways involved in the regulation of cellular senescence [64]. p53 is a transcription factor, and by binding the promoter region of specific target genes, it regulates or downregulates their expression. The p53-dependent modulation of the expression of these and other target genes is implemented by several downstream effectors. Fisher et al. [65] proposed approximately 3661 potential p53 targets. This very high number is the reflection of the numerous roles, and in some cases, even alternative ones, played by p53. These target genes are involved in the regulation of DNA damage repair, autophagy, metabolism, cell cycle arrest, quiescence, senescence, and apoptosis [66]. The results of numerous studies show that p53 plays a very important role in maintaining genomic integrity by being involved in the response to DNA damage. When p53 function is lost, chromosomal instability is promoted (directly and indirectly), causing cells to enter senescence or apoptosis [66]. All this evidence collectively shows that p53 plays a fundamental role in determining cell fate. In the context of senescence, p53 activation can occur in a DNA damage response-dependent or independent manner [67]. There is evidence that these different p53-mediated programs are associated with different p53 levels and that the stressor intensity threshold between senescence and apoptosis differs by cell type, although for some cells, p53 always appears to trigger senescence [66]. The activity of p53 does not only depend on its concentration. It seems that alternative splicing of p53 mRNA and posttranscriptional modifications of p53 protein also play an important role [68]. The microenvironment, p53 concentration, and posttranslational modifications are therefore responsible for inducing precise patterns of gene expression capable of inducing senescence. A much-studied posttranslational modification is the case of a single acetylation on p53. Knight et al. have shown how p53 acetylation induces the activation of genes with high affinity for p53, such as cyclin dependent kinase inhibitor 1A (*CDKN1A*), which encodes p21cip1, and seems to play a key role in the pathway of senescence. If multiple sites are acetylated, hyperphosphorylation of p53 occurs, leading to the interaction with genes with low affinity for p53, such as proapoptotic genes [69]. p21cip1, a member of the mammalian cyclin dependent kinase inhibitor family, has a necessary role in p53-induced cell cycle arrest at the G1/S or G2/M checkpoints. Activated p21cip1 modulates gene expression of many p53 targets such as CDC25B, CDC25C, and Survivin [70].

However, the key role of p21cip1 lies in its ability to promote senescence by inhibiting apoptosis by binding to various apoptotic agents, such as many caspases.

The p53/p21cip1 pathway plays a key role in the initiation of senescence, whereas the p16 and retinoblastoma families of protein pathways appear to play an important role in the maintenance of senescence. These suggestions follow from observing a decrease in p53 levels after induction of senescence, whereas p16 levels remained high [71]. In subsequent studies, Beauséjour et al. [64] showed that p53 had different effects in senescent cells depending on p16 activity, inducing replication and growth in cells with low p16 levels but not in cells with high p16 activity. The results of these studies show that the activation of the p16 pathway draws a dividing line between 2 different phases of senescence: an early reversible phase induced by p53 and an irreversible phase induced by the p16/Rb pathway.

It also appears that the p53/p21cip and p16INK4a/Rb pathways constantly interact and influence each other via different crosstalk mechanisms, which is worthy of further elucidation. It has been observed, for example, that the induction of senescence can be prevented by inactivating p53 before p16 is upregulated, but when p16 is highly expressed, p53 downregulation is not associated with the reversal of cell cycle arrest [72]. Consequently, in another set of experiments, when p53 and pRb were restored to normal levels in human cervical cancer cells, cell senescence was induced in almost all cells [73]. In 2013, Etienne-Selloum et al. [74] described the potential regulation by (poly)phenols of p53 expression at the transcriptional and posttranslational concentration, especially in (poly)phenol-mediated apoptosis of cancer cells. Different (poly)phenols such as curcumin, resveratrol, and procyanidin C1 exert an antisenescence effect through the modulation of p53/p21cip1 and p16INK4A/Rb tumor suppressor pathways [[75], [76], [77]].

### Nuclear factor erythroid 2-related factor 2 (Nrf2) and nuclear factor kappa B (NF-κB)

The multifunctional regulator of nuclear factor erythroid 2-related factor 2 (Nrf2; encoded by the *Nfe2l2* gene) is a transcription factor capable of regulating cellular redox balance and phase II detoxification and protective antioxidant responses in mammals. Nrf2 plays a role in controlling the basal and induced expression of a number of antioxidant response element (ARE)-dependent genes, including NAD(P)H-quinone oxidoreductase 1, glutamate-cysteine ligase, heme oxygenase 1, and thioredoxin reductase 1, which bind to the ARE binding sequence [78]. Nrf2, under homeostatic conditions, is bound to the inhibitory protein Kelch-like ECH-associated protein 1 (Keap1) in the cytosol. However, the conformational alteration of Keap1, induced by different oxidants and electrophiles, determines the release of Nrf2 and its translocation into the nucleus where it binds to the ARE. These steps activate a series of cascading events that ultimately affect the oxidative state of cells, providing important protection against oxidative stress. Nrf2 is not only a cytoprotective factor capable of regulating the expression of genes encoding antioxidant, anti-inflammatory, and detoxifying proteins, but it is also an important modulator of species longevity. Nrf2 is considered “a guardian of the duration of health and guardian of the longevity of the species” [78]. A study conducted on 10 species of rodents, having a maximum potential lifespan between 4 and 31 y, showed that in long-lived naked mole rats, there was a constitutively high concentration of Nrf2 compared with that of other species of rodents having a shorter lifespan [79]. In recent years there has been a growing interest in nutraceuticals capable of stimulating endogenous antioxidant enzymes. It has been observed that some food-active principles such as resveratrol, curcumin, and epicatechins promote cell survival by improving the activity of Nrf2 and the expression of cytoprotective enzymes related to this pathway [80].

### Sirtuins and Klotho

Silent mating type information regulation 2 homolog (SIRT)1 and Klotho are both genes involved in delaying cellular senescence and prolonging lifespan by regulating multiple antiaging cellular processes [[81], [82], [83], [84]]. Sirtuin 1 (Sirt-1), also called NAD-dependent sirtuin deacetylase 1, is encoded by the *SIRT1* gene in humans and belongs to the sirtuin protein family [85]. Sirt-1 deacetylates proteins involved in antiaging mechanisms and various reactions to stressors. Sirt-1 suppresses cellular senescence primarily by delaying age-related telomere attrition, maintaining genome integrity, and promoting DNA damage repair [86]. Sirt-1 also modulates lifespan by interacting with several signaling pathways involved in regulating lifespan, such as the insulin/insulin growth factor-1 (IGF-1) signaling pathway, AMP-activated protein kinase, and forkhead box O (FoxO) [[85], [86], [87]].

Several studies also suggest that some sirtuins are involved in telomeric regulation for their nuclear localization [88,89]. Furthermore, some sirtuins such as Sirt-1 have been observed to interact with some transcription factors related to the hTERT promoter [90] and the C-terminal deacetylates of c-Myc, further influencing the activity of the hTERT promoter [91].

In summary, sirtuin activation improves longevity by modulating key molecules in aging processes, such as FOXO3, Nrf2, and peroxisome proliferator-activated receptor-gamma coactivator 1-alpha, which favor mitochondrial biogenesis and β-oxidation of fatty acids, interact with telomerase transcriptional factors like the hTERT promoter and c-Myc, increase DNA repair processes, delay cell aging by stimulating autophagy, and modulating telomerase activity, cell cycle, and apoptotic processes [92]. In humans, Klotho is an enzyme encoded by the *KL* gene. Klotho, involved in many biological pathways in humans and animals, appears to perform important activities across the lifespan, affecting longevity, memory, cognition [[93], [94], [95]], and kidney function and slowing the progression of some diseases such as diabetes and cancer [84,[96], [97], [98]].

*Klotho*^*-/-*^ mice display a phenotype characterized by premature aging, with a significantly reduced lifespan associated with multiple disorders very similar to those affecting humans such as vascular and renal diseases, atherosclerosis, cognitive impairment, osteoporosis, and infertility [96,99]. Furthermore, during aging, the expression of the *Klotho* gene is reduced in the kidneys and liver [100,101].

A topic widely discussed and analyzed in antiaging research is precisely the search for Sirt-1 and Klotho activators. Recently, some in vivo studies have shown how resveratrol can modulate the signaling pathways mediated by Sirt-1 [[102], [103], [104]]. Other (poly)phenols such as quercetin, naringenin, and silymarin reversed the age-related impairment of monoaminergic neurotransmitter secretion by increasing Sirt-1 levels and inhibiting NF-κB in the rat hippocampus, restoring motor coordination and cognitive functions [105].

### FoxO family

The forkhead box O3 gene (*FOXO3*) belongs to a family of transcription factors (FoxOs) that are associated with the regulation of genes involved in DNA protection, oxidative stress, inflammation, and metabolism [106,107]. FoxO factors are regulated by several factors involved in transduction cascades. One of the major regulators of FoxO function is the phosphoinositide 3-kinase pathway, with FoxO function “tuned” by protein casein kinase 1 and the dual specificity regulated kinase 1A pathway. By phosphorylating FoxO factors within several domains, the kinases regulate the intracellular localization and function of FoxO proteins. In longevity, the role of FOXO3 appears directly linked to the ability of this transcription factor to regulate some cellular functions, such as resistance to oxidative stress, DNA damage, autophagy, metabolism, and programmed cell death. One of the key factors in mitigating aging and age-related diseases, including cardiovascular disease, neurodegenerative diseases, type II diabetes, and various cancers, is the FOXO3 response to environmental stimuli. Indeed, FOXO3 appears to be a potential target for possible therapeutic interventions aimed at promoting healthy aging and prolonging life span. *FOXO3* would appear to be one of the key genes in the insulin/IGF-1 pathway, capable of influencing lifespan in several species. In humans, several single nucleotide polymorphisms in the *FOXO3* gene have been associated with longevity in several populations [106,107]. A link between the insulin/IGF-1 signaling pathway and longevity was first identified in the nematode *C. elegans*, demonstrating how the effect of mutations in DAF-2 (homologous insulin receptor) on life span depends on its effector, DAF-16, an ortholog of human FOXO [108]. Calorie restriction, considered one of the most effective strategies to promote healthy aging and longevity, significantly activates DAF-16 and FOXO3, thereby negating the longevity-enhancing effect of calorie restriction [109]. Nutraceutical compounds such as curcumin, resveratrol, and astaxanthin could therefore help to improve health and increase lifespan by inducing FOXO3.

### Molecular mechanisms by dietary (poly)phenols in senescent and cancer cells

Progress in scientific research in plant phytocomplexes has identified several phytochemical compounds with antiaging potential through different mechanisms, including inhibition of metabolic pathways mediated by IGF-1 and mechanistic target of rapamycin (mTOR); free oxygen and nitrogen radicals scavenger action; inhibition of proinflammatory transcription factors such as NF-κB; stimulation of molecules involved in DNA repair such as poly (ADP-ribose) polymerase 1, FOXO3, sirtuins, and Nrf2; and maintenance of telomere length [110]. Among these, (poly)phenols such as curcumin, resveratrol, ECGC, quercetin, and fisetin [111] have lately received special attention and are here discussed.

### Curcumin

Curcumin is the main polyphenolic compound extracted from the rhizomes of *Curcuma longa* (known as turmeric). Long-term studies have shown that curcumin intake ranges from 40 mg to 8 to 12 g/d, with higher doses usually used for cancer studies, whereas the lower doses, which are achievable through diet, are commonly used to test curcumin’s antioxidant and anti-inflammatory mechanisms [[112], [113], [114]]. Up to 12 g/d of curcumin use seems safe and potentially reduces risk of several inflammatory and age-related diseases as well as various cancers [114,115]. Our group, together with others, demonstrated that some of curcumin’s beneficial effects can be both related to its capability to modulate acute and chronic inflammation by scavenging ROS because of the electron-donating group on curcumin’s phenolic rings or to curcumin metabolites produced by its interaction with gut microbiota, which can explain the very low curcumin concentrations detected in urine and plasma after oral administration in both animal and human studies [[116], [117], [118], [119]]. Observations on curcumin kinetics reveal markedly low serum levels, rapid metabolism with the formation of inactive metabolites, limited tissue distribution, and rapid clearance/elimination from the body [114]. However, different biological activities of curcumin have been reported in human studies [[120], [121], [122], [123]]. The current debate to untangle the ambiguity between the bioavailability and biological activity of curcumin also seems to reside on curcumin’s bidirectional interaction with gut microbiota and an individual’s metabolic capacity, which depends on microbiota biodiversity [124]. However, for cancer treatments in particular, where higher curcumin dosages are required, novel curcumin formulations such as incorporation into micelles, micro/nanoemulsions, nanoparticles, and liposomes are currently being developed to enhance curcumin bioavailability [114].

**FIGURE 2:**
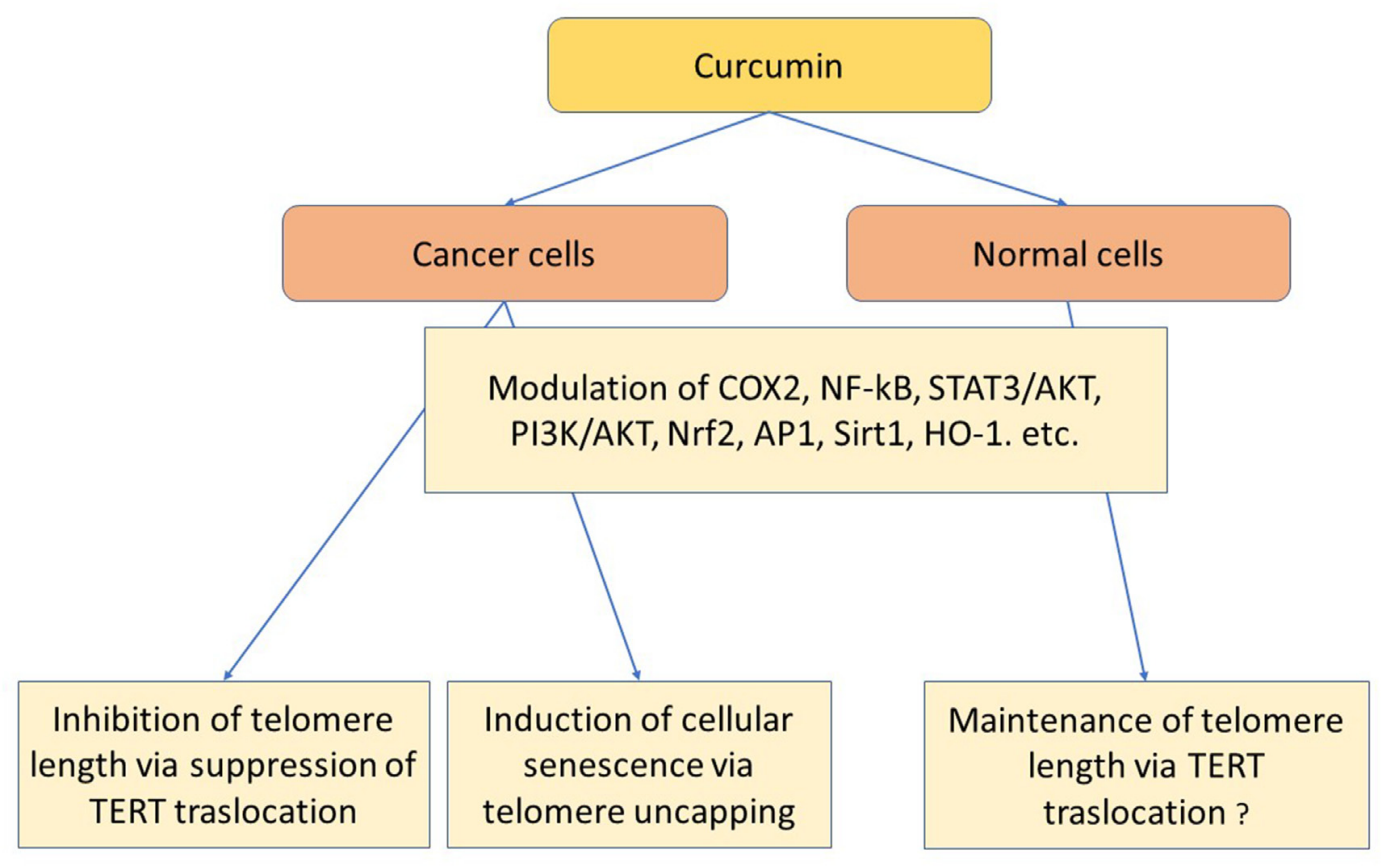
Anticancer and longevity mechanisms of curcumin. AKT, protein kinase B; AP1, activator protein 1; COX-2, cyclooxygenase 2; HO-1, heme oxygenase 1; NF-κB, nuclear factor kappa B; Nrf2, nuclear factor erythroid 2-related factor 2; PI3K, phosphatidylinositol 3-kinase; Sirt1, sirtuin 1; STAT3, signal transducer and transcription activator 3; TERT, telomerase reverse transcriptase.

Cancer cells possess deregulated signaling pathways related to oxidative stress, proliferation, inflammation, and angiogenesis [125,126], and curcumin’s anticancer properties may not be limited to its anti-inflammatory and redox-modulating mechanisms [127]. Indeed, curcumin is known to act on multiple signaling pathways and recognizes a plethora of molecular targets involved in cancer development [128] including cyclooxygenase (COX)-2, cytokines, transcription factors like protein kinase B, NF-κB, signal transducer and transcription activator 3 (STAT3), activator protein 1, and telomerase [129,130]. In particular, telomerase modulation represents a potentially selective target for both cancer and antiaging therapy, and curcumin seems to both inhibit and maintain telomerase activity in vitro in a time- and dose-dependent manner [131,132] through regulation of TERT translocation from the cytosol to the nucleus [133] (Figure 2).

Curcumin has additionally been shown to stabilize Nrf2 thus triggering the Nrf2 pathway, leading to downstream activation of antioxidative enzymes including heme oxygenase, Hsp70, thioredoxin reductase, and sirtuins. On the contrary, high curcumin concentrations can induce a prosenescence role in cancer cells [134,135].

Interestingly, curcumin was shown to inhibit cell growth in a concentration-dependent manner and suppress telomerase activity in human tumor cell lines [131]. In another study in the human leukemia cell line K-562, curcumin inhibited telomerase activity in a dose- and time-dependent manner through the suppression of TERT translocation from the cytosol to the nucleus. Curcumin inhibition of telomerase activity correlated with several apoptosis-associated parameters, suggesting that telomerase status plays an important role in curcumin induction of apoptosis in K-562 cells [132]. In some other studies in tumor cells, long-term treatment with a high dose of curcumin induced cellular senescence via telomere uncapping [[136], [137], [138]]. In conclusion, because most of the studies that link curcumin with telomeres and telomerase are aimed at anticancer therapies, it would be appropriate to design new studies analyzing the action of curcumin on telomerase activity and telomere length in the antiaging context.

On the other hand, no direct evidence showing the antiaging effect of curcumin on normal cellular phenotypes related to telomere maintenance and telomerase activity has been reported (Figure 2). It is known that curcumin activates the phosphoinositide 3-kinase (PI3K)/protein kinase B (AKT) pathway, which induces FOXO-dependent gene expression, which is related to decreased oxidative stress and increased life span. Curcumin also modulates multiple factors involved in inflammation and apoptosis such as caspase 9, Bad, NF-κB, Fast, and Bcl-2. Inhibition of the mTORC1 pathway and activation of Nrf2 are other mechanisms by which curcumin could increase the lifespan of normal cells. Recent data have indeed shown that Nrf2 interacts with X-ray repair cross complementing 5 and regulates hTERT expression, increasing chromosome integrity and genome stability for long-term proliferation [139]. Overall, a multitude of in vivo, in vitro*,* and clinical studies have confirmed the protective role of curcumin via Nrf2 regulation, which in turn could maintain telomere integrity [118,140].

Longevity is also associated with other signaling pathways related to telomere protection, like the homeostasis master regulator FOXO3. A recent study demonstrated a direct connection between FOXO3 and telomere protection, highlighting an unexpectedly higher level of integration in the regulation of the longevity signaling pathway [141].

Different natural compounds favor FOXO3-mediated antiaging mechanisms [142,143]. Curcumin reduces FOXO3 phosphorylation, leading to nuclear translocation and a 2-fold increase in FOXO3-mediated gene expression, thereby protecting against oxidative and lipid-induced damage in inflammatory cells and reducing risk of age-associated diseases [106].

### Resveratrol

Among several potential geroprotectors, resveratrol is considered to be one of the most interesting. Resveratrol is the 3,5,4’-trihydroxy *trans*-stilbene, a phytoalexin used by the plant as a defense response to various stress conditions. This compound, known for its potential protective role against degenerative diseases, is synthesized by numerous plants such as *Polygonum cuspidatum* and is present in considerable quantities in grapes and red wine. In *Polygonum cuspidatum*, it is present in glycosylated form and as a *trans* isomer that is known to favor absorption and biological activity. After oral ingestion, human resveratrol absorption is ∼75%, mainly occurring by transepithelial diffusion. However, resveratrol oral bioavailability is <1% due to extensive metabolism in the intestine and liver by endogenous enzymes and by the gut microbiota. Metabolic studies, both in urine and plasma, have revealed extensive metabolism of resveratrol in the liver and intestine operated by endogenous enzymes where the major metabolites are glucuronides and sulfates [144]. In humans, sulfation was recently considered more important than glucuronidation [144,145]. However, reduced dihydroresveratrol conjugates, in addition to highly polar unknown products, may account for as much as 50% of an oral resveratrol dose. The gut microbiota metabolizes resveratrol to dihydroresveratrol, lunularin, and 3,4′-dihydroxy-*trans*-stilbene, with high interindividual variability in the type of metabolites produced, which are biologically active [146].

Numerous in vitro, in vivo*,* and clinical studies, have suggested its potential use as a regulator of cardiovascular system functionality [147], bronchial secretion fluidity, and body fluid drainage [148] in counteracting oxidative stress [149,150] and physical and mental fatigue [151], in promoting menstrual cycle regularity, and as an antiaging molecule [152,153]. The latter effect seems to be achieved through the activation of longevity-associated factors, like sirtuins, demonstrated in *C. elegans*, *D. melanogaster*, and *S. cerevisiae* [154] as well as in mammals [155].

**FIGURE 3:**
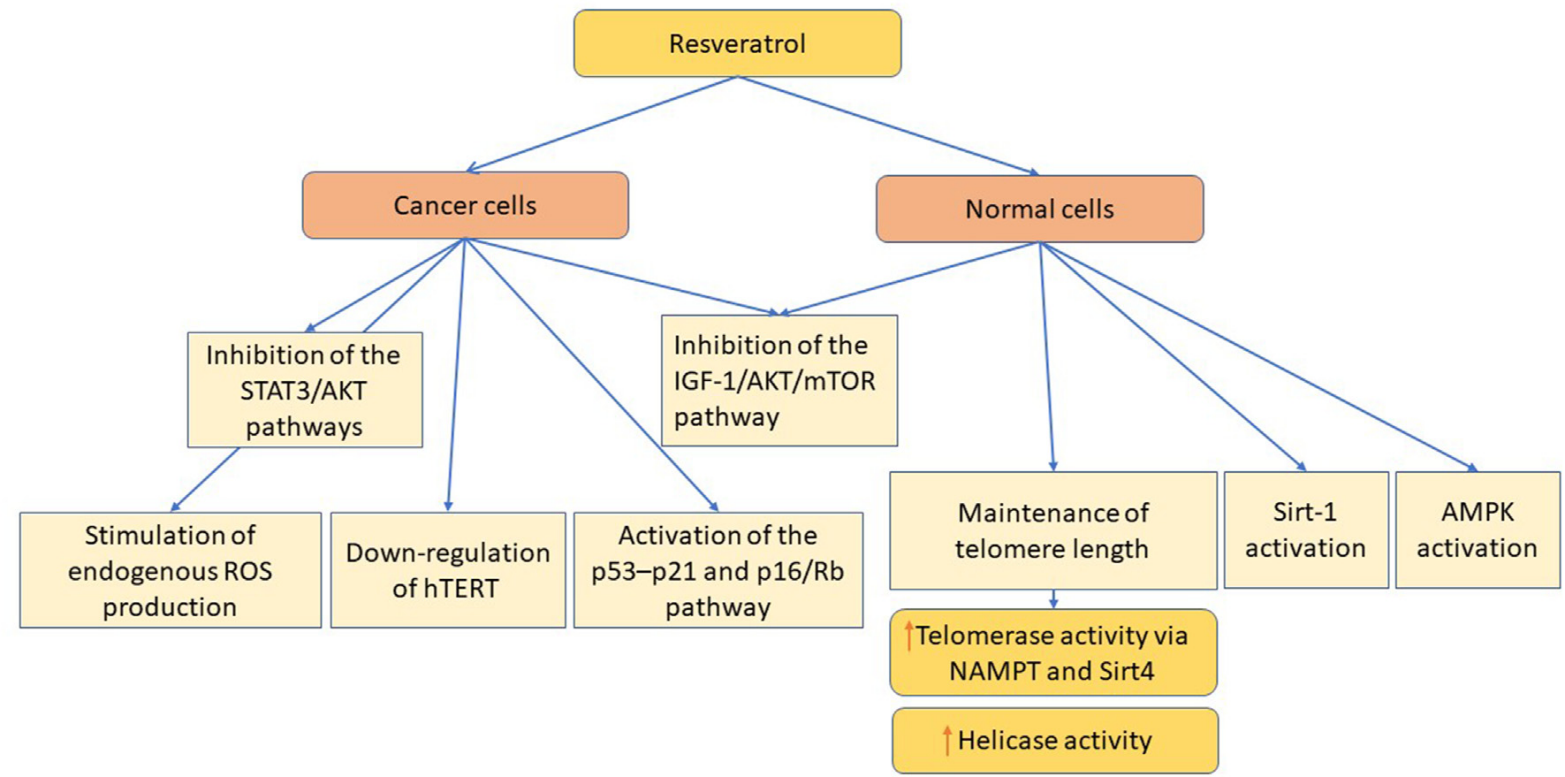
Anticancer and longevity mechanisms of resveratrol. AMPK, 5’ adenosine monophosphate-activated protein kinase; AKT, protein kinase B; hTERT, human telomerase reverse transcriptase; IGF-1, insulin growth factor 1; mTOR, mechanistic target of rapamycin; NAMPT, nicotinamide phosphoribosyltransferase; ROS, reactive oxygen species; Sirt1, sirtuin 1; Sirt4, sirtuin 4; STAT3, signal transducer and transcription activator 3.

Moreover, resveratrol induces the activation of the 5’ adenosine monophosphate-activated protein kinase (AMPK), a key enzyme in energy metabolism. AMPK is a cellular energy sensor that is physiologically activated by physical exercise, which modulates, among other things, appetite and insulin metabolism through the induction of glucose transporter translocation on the cell membranes, thus reducing blood glucose levels. Further, AMPK in turn modulates sirtuin activation and inhibits the IGF-1/AKT/mTOR signal pathway involved in aging (Figure 3) [92].

In addition, resveratrol has been shown to promote telomerase activity in vitro, helping to maintain telomere length by acting on telomerase and helicase. Moreover, resveratrol has the potential to attenuate telomere attrition by inducing enzyme repair systems [156]. Resveratrol induces the activation of helicase and telomerase without affecting cell proliferation [157]. Further, resveratrol delays the senescence of endothelial progenitor cells through telomerase activation and Akt phosphorylation [158,159]. Recently, resveratrol has also been shown to influence antiaging processes in human aortic smooth muscle cells by activating human telomerase through nicotinamide phosphoribosyltransferase and sirtuin 4 (SIRT4), thus suggesting a new mechanism underlying the beneficial effects of resveratrol on cardiovascular disease and aging processes [160]. A recent review analyzed the effects of resveratrol on the cardiovascular system and its potential beneficial use in the prevention of long-term cardiovascular morbidity and mortality. Focusing on factors that contribute to postmyocardial infarction remodeling and influence aging and telomere length, the review highlighted a protelomerase effect of resveratrol that could counteract the development of negative postmyocardial infarction remodeling [161].

Numerous studies have also shown the anticancer effect of resveratrol and its inhibitory activity on telomerase and telomere length [162,163]. Chung et al. [164] found that the combination of 5-fluorouracil and resveratrol enhances antitelomerase activity and induces apoptosis by inhibiting the Akt and STAT3 signaling pathways in human colorectal cancer cells, leading to resensitization to chemotherapy. Resveratrol has also been shown to induce senescence of breast cancer cells through the p53–p21 pathway [75], a mechanism, together with the p16/Rb pathway, associated with senescence and long-term cell cycle arrest in cancer cells [75]. Stimulation of endogenous ROS production by mitochondria has also been shown to play a key role in the resveratrol induction of senescence in cancer cells [165]. Finally, Kala et al. [166] found that the association of resveratrol and pterostilbene resulted in synergistic growth inhibition of triple-negative breast cancer cells.

In normal cells, SIRT1 protects cells against environmental stress and potential carcinogenic agents. SIRT1 is a key factor in the resveratrol activation of AMPK and mitochondrial function both in vitro and in vivo [167]. However, in malignant growth, it contributes to gene silencing and initiation and/or maintenance of cancer through the overproduction of the telomerase enzyme, which maintains cancer cell replication indefinitely [168]. As >90% of human cancers overexpress hTERT, but not normal somatic cells do not, it seems that resveratrol and pterostilbene selectively induce hTERT downregulation at both the gene and enzymatic activity levels [166]. Targeting SIRT1 and hTERT has thus emerged as promising strategies for cancer therapeutics [165].

In human trials, the anticancer and senolytic activity of resveratrol is achieved with high doses, ≤5 g/d [169]. Notably, relevant amounts of resveratrol can be obtained only through concentrated supplements because its concentration in the human diet is limited to a few foods that contain low resveratrol levels, including grapes, peanuts, red wine, some types of berries, and pistachios [146].

### Epigallocatechin gallate (EGCG)

Green tea is produced by processing the leaves of *Camellia sinensis* (Theaceae). EGCG and chlorogenic acid, 2 important phenolic compounds in green tea, are believed to be responsible for most of its biological effects. Above small-intestinal absorption, tea (poly)phenols could reach the large intestine and be processed by gut microbiota mainly into gallic acid and epigallocatechin [170]. Several epidemiological and clinical studies have highlighted the anticarcinogenic effects of green tea (poly)phenols on various cancers such as breast, colon, lung, and blood cancers but also its preventive effects on age-related diseases [[171], [172], [173]].

Several studies have described green tea polyphenol effect on telomere modulation in cancer cells, analyzing the antioxidant and pro-oxidant, anti-inflammatory, and antiangiogenic effects; the induction of apoptosis; the modulation of epigenetic pathways; and EGCG binding to cancer-related proteins [[173], [174], [175], [176], [177]].

**FIGURE 4:**
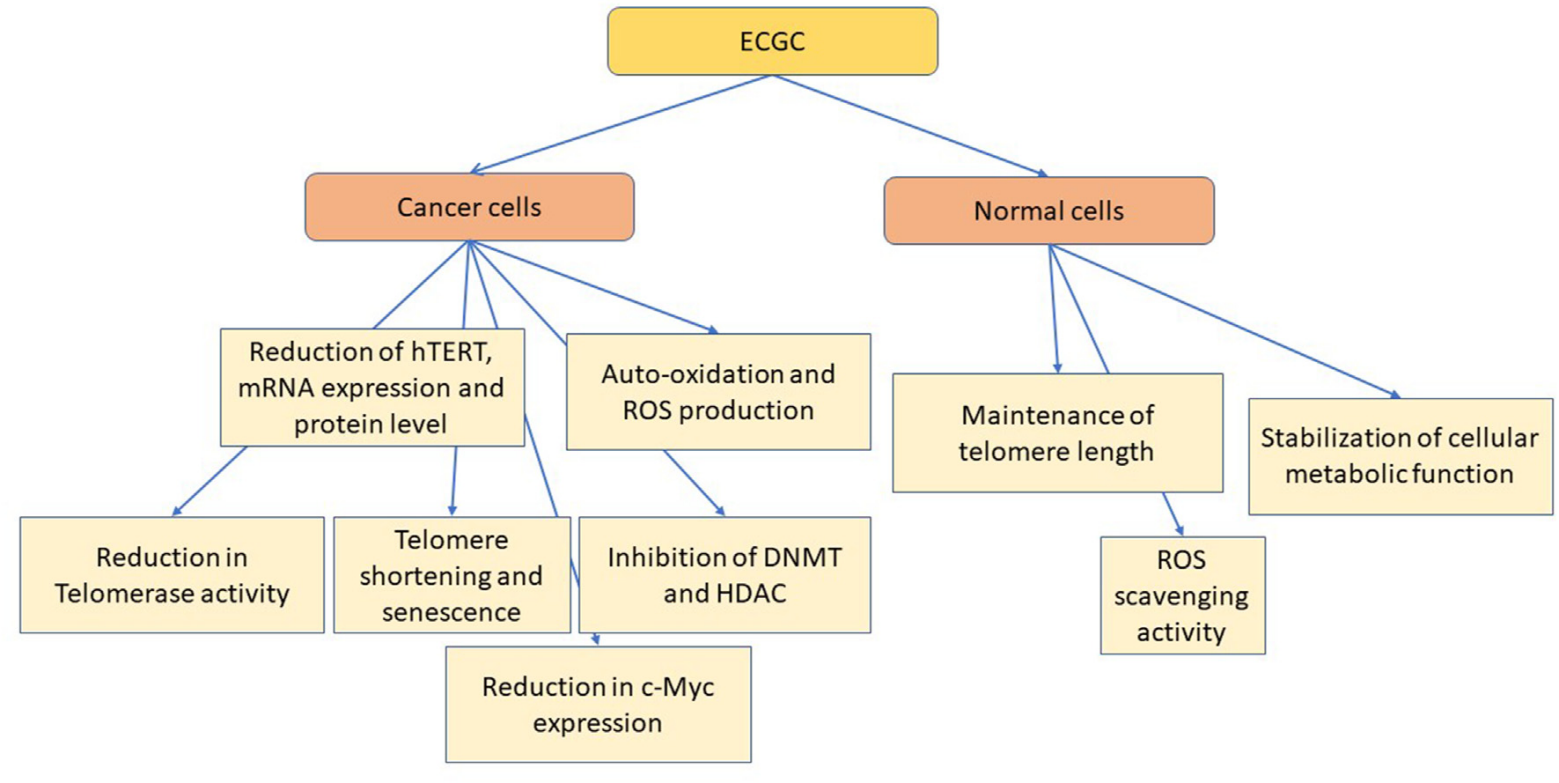
Anticancer and longevity mechanisms of ECGC. DNMT, DNA methyltransferase; HDAC, histone deacetylases; hTERT, human telomerase reverse transcriptase; ROS, reactive oxygen species.

EGCG has specific and direct anticancer activity on some tumor cells, selectively regulating cancer signaling pathways (Figure 4) [178].

A dose-dependent association was indeed observed between EGCG and telomere length in normal cells and, conversely, a decrease in telomerase and hTERT gene expression levels in tumor cells, supporting the hypothesis that EGCG has selective antiproliferative effects on cancer cells [179,180]. In vitro treatment of human breast carcinoma MCF-7 cells with EGCG dose-dependently inhibited telomerase activity, as well as hTERT mRNA expression, without affecting telomerase activity in normal mammary cells [181]. Additional studies further demonstrated that EGCG inhibited hTERT protein expression, thus confirming that inhibition of telomerase was associated with the downregulation of hTERT [182,183]. In particular, EGCG has been tested on normal, nonmalignant, and cancerous cells to understand the epigenetic mechanisms involved in ECGC chemopreventive activities. Treatments with EGCG and a novel prodrug of EGCG dose- and time-dependently inhibited in vitro the proliferation of human breast cancer MCF-7 and MDA-MB-231 cells but not normal control MCF10A cells. In particular, EGCG and pro-EGCG both inhibited the transcription of *hTERT* through epigenetic mechanisms in estrogen receptor-positive MCF-7 and estrogen receptor-negative MDA-MB-231 cells [184]. In summary, EGCG acts on hTERT and inhibits the catalytic function of telomerase promoting telomere shortening and senescence in human cancer cells [185]. EGCG has been found to act as an epigenetic regulator in cancer cells by inhibiting 5-cytosine DNA methyltransferase [186], histone deacetylase [187], and telomerase activity, thus arresting tumor proliferation [180]. Hypermethylation of the *hTERT* gene was correlated with telomerase activity in both healthy and tumor tissues, whereas, conversely, demethylation was not associated with increased hTERT expression [188]. Although several transcription factors and pathways are involved in the regulation of hTERT expression, the transcriptional repressor CTCF appears to be a key determinant related to mortality [189]. Taken together these results underline the impact that EGCG may have on telomere regulation and cellular aging.

c-Myc, whose function is associated with the expression of hTERT, is considered the main regulator of tumorigenesis because it controls many aspects of cell proliferation, differentiation, and cell metabolism [190]. Activation of c-Myc has been observed in more than half of human tumors, and its dysregulation is considered a marker of genomic instability [191]. The potential EGCG effect on c-Myc expression was investigated in several cell lines, and a significant decrease in c-Myc expression together with reduced levels of hTERT proteins after EGCG treatment was observed, an activity possibly mediated by NF-κB [192]. Methylation of c-Myc is considered an indirect mechanism used to regulate telomerase through hTERT. In fact, it has been observed that c-Myc methylation is significantly higher in some tumor cell lines after treatment with EGCG [193]. The methylation status of hTERT was, in some cancer cell lines such as Caco-2 cells, little affected by EGCG treatment [193]. Conversely, in other tumor cell lines, such as HeLa cells (cervical tumor cell line) a significant decrease in telomere length and decreased telomerase activity was observed after treatment with EGCG [182]. Based on these results, it was suggested that telomere shortening could be further enhanced by EGCG-mediated telomerase inhibition through ROS generation, telomeric DNA being particularly susceptible to ROS-mediated damage along with telomeric attrition [194]. The biological effects of EGCG are dose-dependent, and at concentrations between 10 and 200 μM EGCG, cause antiproliferative effects in some cultures of human tumor cells [[195], [196], [197]], whereas at physiological concentrations of EGCG (<10 μM), a stabilization of the cellular metabolic function is observed [195,198], thus suggesting that the anticancer effect of EGCG requires higher doses.

In other studies, EGCG auto-oxidation was observed to produce ROS in a dose-dependent manner [199], with low concentrations (<5 μM) promoting cell growth-favorable conditions while inducing ROS production, inhibition of telomerase activity, and induction of apoptosis at higher doses (>50 μM) [199,200]. It remains to be clarified whether such self-oxidative properties of EGCG can also occur in vivo and what potential toxicological side effects might occur on macromolecules such as proteins or lipids [199]. For this purpose, in some in vitro studies, the levels of malondialdehyde were evaluated to obtain an initial indication of the potential pro-oxidative effects of EGCG on such molecules [201,202]. The antioxidant properties of EGCG are mediated by several mechanisms, including the activation or inhibition of sirtuins, extensively studied for their relevance in cancer biology and age-related diseases [203].

Finally, microRNAs modulate the expression of several oncogenes and tumor suppressor genes including the genes that regulate tumor angiogenesis. Recent evidence indicates the interaction of microRNAs with hTERT in the regulation of telomerase [204]. EGCG seems to have the ability to globally inhibit inflammatory responses via modulation of microRNA expressions, thus indirectly affecting cell aging and cancer cells [205,206].

### Quercetin and fisetin

Quercetin (3,3,4,5,7-pentahydroxyflavone) is a flavonoid polyphenol widely occurring in nature as a plant secondary metabolite, found abundantly in different vegetables and dietary products, notably onions, apples, and red wine [10,207]. It has been estimated that humans consume 10–100 mg quercetin every day on average through their diet [[208], [209], [210]]. However, quercetin dosages most often used in clinical trials range from 200 to 1200 mg by mouth daily for ≤12 wk [208,211]. Numerous studies have shown several potential clinical applications for quercetin such as the treatment of inflammation, allergies, arthritis, cancer, cardiovascular disorders, and age-related diseases, including neurodegenerative conditions [212]. Quercetin bioavailability varies depending on the sugar moiety, and some glycosides of quercetin are well absorbed. Increasing evidence in the literature suggests that quercetin’s metabolites may be responsible for the abovementioned health benefits. Three observations support the role of quercetin metabolites in human health: pH-dependent quercetin degradation in the basic environment of the intestines; low absorption quercetin in the intestine, resulting in low bioavailability; and quercetin biotransformation by the resident gut microbiota. The gut microbiota is, in fact, capable of quercetin biotransformation to generate bioactive metabolites such as homoprocatechuic acid, protocatechuic acid, 4-hydroxybenzoic acid, and 3-(3-hydroxyphenyl)propionic acid, which are, in part, responsible, for example, for quercetin antiproliferative activity [213,214]. Quercetin is one of the most pleiotropic and versatile natural flavonoids, without any notable toxicity to date, favoring application for long-term preventive treatments for a wide array of clinical indications.

**FIGURE 5:**
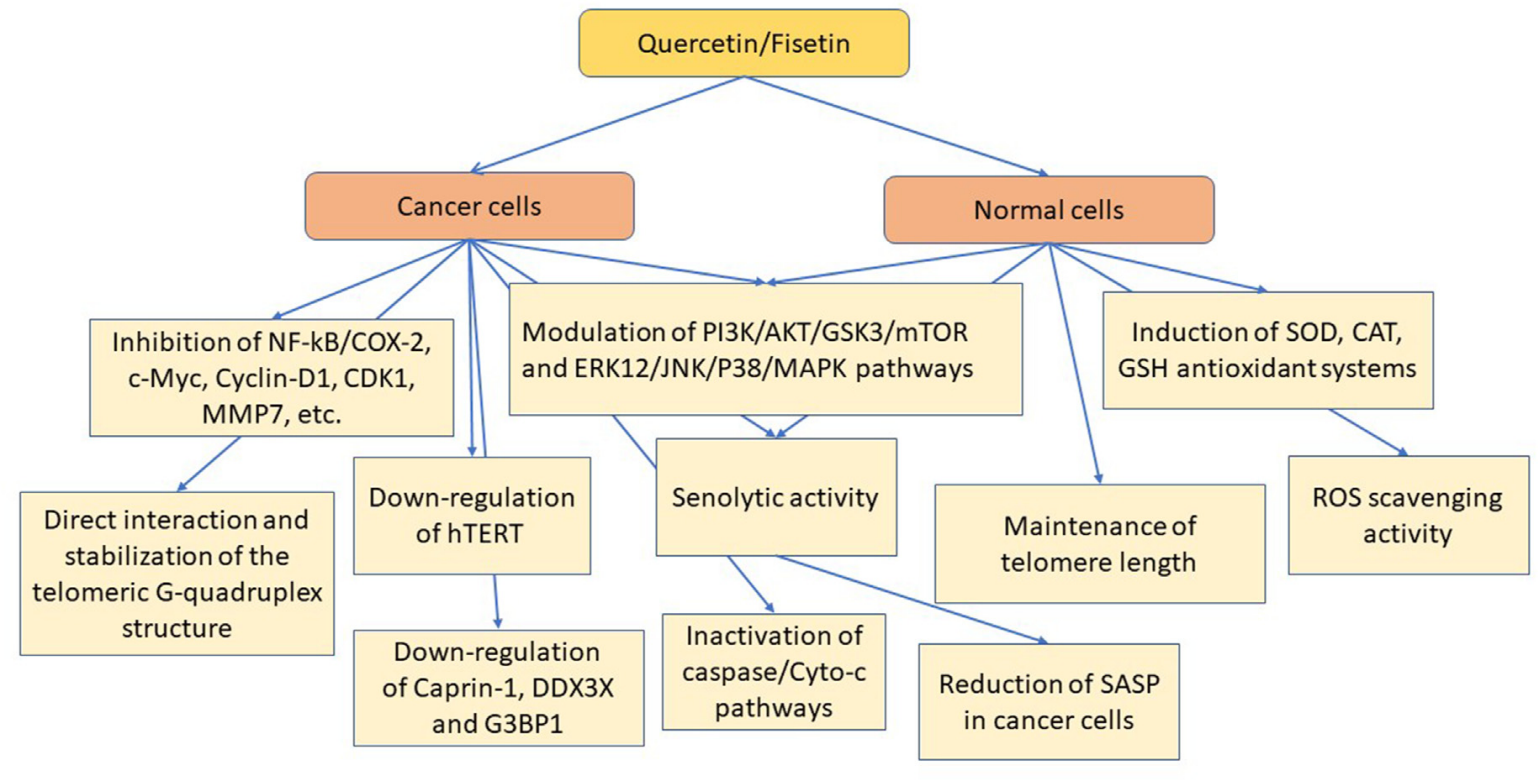
Anticancer and longevity mechanisms of quercetin and fisetin. AKT, protein kinase B; CAT, catalase; CDK1, cyclin dependent kinase 1; COX-2, cyclooxygenase 2; DDX3X, DEAD-box helicase 3 X-linked; ERK1/2, extracellular signal-regulated kinase 1/2; G3BP1, Ras-GTPase-activating protein (GAP)-binding protein 1; GSH, glutathione; GSK3, glycogen synthase kinase 3; hTERT, human telomerase reverse transcriptase; JNK, c-Jun N-terminal kinase; MAPK, mitogen-activated protein kinase; MMP7, matrix metalloproteinase 7; mTOR, mechanistic target of rapamycin; NF-κB, nuclear factor kappa B; PI3K, phosphatidylinositol 3-kinase; ROS, reactive oxygen species; SASP, senescence-associated secretory phenotype; SOD, superoxide dismutase.

In addition to its flavonoid chemical structure that endows quercetin with strong antioxidant and free radical scavenging activity, quercetin has been shown to positively regulate elements of the antioxidant enzymatic balance (i.e., superoxide dismutase, catalase, and glutathione) (Figure 5). This confers quercetin a particularly effective antioxidant potential, exploitable for the treatment and prevention of several chronic and degenerative clinical conditions, especially those associated with aging [212]. The potential of quercetin in aging-associated conditions is also reinforced by its direct activity on cellular proinflammatory pathways like P13K/AKT/glycogen synthase kinase 3 and extracellular signal-regulated kinase 1/2 (ERK1/2)/c-Jun N-terminal kinase (JNK)/P38 mitogen-activated protein kinase (MAPK), as well as key regulators like inducible nitric oxide synthase and NF-κB, leading to downregulation of cytokines, thus modulating the self-amplifying inflammatory cascade [212], characteristic of chronic and degenerative conditions, like those induced by SASP.

Numerous studies have focused on quercetin anticancer activities, and various antitumoral molecular mechanisms have been highlighted in cancer cells, from induction of apoptosis and reduced proliferation to cell cycle arrest and inhibition of metastasis [[215], [216], [217]]. Multiple targets have been demonstrated in cancer cells, including modulation of NF-κB, sirtuins, cyclins, and other proteins involved in cell cycle regulation, heat shock protein 70, antiapoptotic proteins coded by Bcl-xL and Bcl-2, and cytochrome c oxidase subunit 2, p-JNK/c-Jun axis, PI3K/Akt/mTOR pathway, as well as upregulation of proapoptotic factors like p53, p21, Bid, Bax, Bad, cytochrome c, caspase-3, and caspase-9 [[217], [218], [219], [220], [221], [222]].

An extensive proteomic analysis was also carried out to better identify pathways involved in the early stages of quercetin-induced apoptosis, using a cellular model of human chronic myeloid leukemia (K562 cells). A significant modulation could be shown in the antioxidant defense systems and the cellular translational machinery involved in cell death and survival, as well as a downregulation of Caprin-1, DEAD-box helicase 3 X-linked, and Ras-GTPase-activating protein (GAP)-binding protein 1, 3 proteins involved in cell cycle progression, and upregulation of Annexin 1, a membrane-binding protein known to be associated with early events of apoptosis [223].

Using a systems biology approach, quercetin emerged as the main active ingredient of a traditional medicine decoction, Baiying Qinghou, used in the treatment of laryngeal squamous cell carcinoma. In the network pharmacology analysis carried out to elucidate the pharmacological mechanisms of action of Baiying Qinghou, quercetin was predicted to be the active compound, targeting the signaling pathway PI3K-AKT and TP53, EGFR, NOS3, IL1 involved in drug resistance [224].

Besides acting on many cellular pathways, quercetin has also been shown to directly suppress telomerase activity in several leukemic cell lines and in chronic myeloid leukemia cells, where it has a strong antiproliferative and apoptotic effect [223,225]. Likewise, in liver cancer cells, quercetin nanoparticles inhibited hTERT by downregulating AP-2β expression and decreasing its binding to the *hTERT* promoter. In the same model, quercetin antitumor activity was demonstrated to be associated with multiple routes of action, bedsides telomerase inhibition also inactivation of caspase/Cyto-c pathway, inhibition of NF-κB/COX-2 and Akt/ERK1/2 signaling pathways, as well as proapoptotic downregulation of c-Myc, cyclin-D1, cyclin dependent kinase 1, matrix metalloproteinase 7, and β-catenin [226]. It has also been proposed that quercetin and other flavonoids’ anticancer activity could be exerted on telomeres by direct interaction and stabilization of the telomeric G-quadruplex structure [227,228]. Other flavonoids and derivatives like the isoflavonoid genistein and the flavonolignan silibinin are known to exert an inhibitory effect on telomerase activity [229].

Given these tumor suppressive effects, quercetin and other flavonoids have recently received special attention, and their effectiveness has been put under scrutiny. Besides their direct effect on cancer cells, one of the most promising approaches aims at exploiting the suppressive effect of these flavonoids on senescent cells [63,230,231]. These cells can create a favorable milieu for the growth and development of cancer, and at the same time, cell senescence of nonmalignant cells can be induced as a negative side effect of chemotherapy or radiation, ultimately interfering with their effectiveness. Quercetin and other flavonoids have been shown to selectively destroy these cells (senolytic effect) effectively rejuvenating tissues, and their association with antitumoral agents has opened new avenues to explore for cancer therapy [232]. In particular, the combination of dasatinib (a tyrosine kinase inhibitor) plus quercetin (D+Q) has been proposed for clinical application in various types of cancer where it acts principally by combining antiproliferating cancer therapy with senolytic effects [63,233,234]. Other combinations of cancer therapies and senolytics are being explored using quercetin derivatives [235] as well as natural compounds, like curcumin and flavonols quercetin and fisetin [236]. Interestingly, fisetin, a flavonoid abundant in strawberries, apples, and persimmons, is more active than quercetin and can achieve the same effects on cancer cells by itself, without the addition of other anticancer agents [232,233]. Like quercetin, fisetin has also been shown to possess other wide-ranging activities on pathways involved in key cellular processes, like apoptosis, cell cycle, inflammation, and angiogenesis, which can reinforce its antitumoral effect [237].

Cellular senescence can be a promoter of age-related diseases and contributes to tissue and organ aging and dysfunction. In general, by modulating pathways involved in senescence, flavonoids are believed to be good candidates for preventing or delaying senescence and the aging process as well as age-associated pathologies. Senolytic flavonoids, like quercetin and fisetin, have been proposed in antiaging therapies, thus opening the way to many clinical applications. Their ability to target selectively senescent cells by eliminating them and mitigating their proaging consequences, as well as downregulating SASPs, has been suggested to improve lifespan [63,238]. The process of senescence activation is closely related to the cell cycle regulatory machinery. The ability of flavonoids to act on key factors in senescence pathways, like cyclins, telomeres, and other pathways activated in the DNA damage response, in addition to their strong antioxidant effect, makes them formidable candidates in antiaging strategies, as well as degenerative diseases associated with age and senescence [63,233,239,240]. Quercetin has been shown to have a positive effect on longevity in various experimental models, and although further investigations are required, studies in *C. elegans* have associated the ROS scavenging activity, believed to be a key component in its antiaging effect, with the MAPK pathways and the downstream target DAF-16/FOXO transcription factor [241]. Quercetin alone or in association with dasatanib has been successfully used to mitigate cell senescence and its effects in various models and has also been subjected to clinical trials in diabetes-associated conditions, Alzheimer’s disease, elderly frail patients, and coronary artery disease, to name a few [63,242]. Data to support quercetin use in aging and age-related conditions are rapidly accumulating. The senolytic effect of quercetin has been demonstrated in various models, from endothelial cells to adipocytes to ovarian aging [240,243,244]. D+Q has been shown to inhibit the tumor necrosis factor receptor associated factor 6/MAPK/NF-κB axis, thus alleviating human umbilical vein endothelial cell senescence, a model of age-related cardiovascular diseases [245]. The same association proved effective in vitro against senescent kidney tubule epithelial cells, thus suggesting a mechanism to prevent renal fibrosis [246]. D+Q has also been suggested to ameliorate type 2 diabetes, where it improves both glucose tolerance and insulin resistance, an effect possibly associated with the reduction of senescent cell populations in adipose tissue, as shown in a model of animals with high-fat-diet-induced diabetes with obesity [239]. D+Q association has been shown to target senescent cells, decrease serum levels of inflammatory mediators of the senescent cell secretome, and alleviate aging-related memory impairment and learning deficits in aged rats [247]. Similarly, in aged rats, D+Q combination improved cognitive function and reduced microglial activation and expression of SASP factors [248]. In a mouse model (SAMP10 mouse strain), used to study brain aging, D+Q improved the frailty index, used to assess the increased vulnerability to stressors and can be considered a measure of risk status in older adults. In the same model, the association was also shown to improve motor and cognitive functions as well as the senescent phenotype of the hippocampus, thus confirming the senolytic effect of the flavonoid [249].

An interesting emerging application for senolytics is SARS-CoV-2 infection, where senescent cells can amplify susceptibility to infection and virus-induced lung inflammation. The D+Q senolytic combination proved effective in a model of SARS-CoV-2-evoked cellular senescence in infected cells, where it selectively eliminated virus-induced senescent cells and mitigated lung inflammation and disease [250]. Similar results with the D+Q combination, as well as with fisetin, were obtained in old mice acutely infected with SARS-CoV-2 where a significant survival was also observed. Clinical trials are underway to validate these promising results [251].

The senolytic effects of quercetin have been demonstrated also in its natural products. Rutin, a glycoside of quercetin, has been shown in in vivo and in vitro models to attenuate diabetic atherosclerosis by inhibiting oxidative stress and protecting telomeres [252]. Phytocomplexes containing quercetin have also been demonstrated to exert antiaging activity, like *Phyllanthus emblica* L. extracted (poly)phenols, which were shown to extend lifespan in the *C. elegans* model [253].

Closely related to quercetin, the natural flavonol fisetin (3,30,40,7-tetrahydroxyflavone) possesses stronger senolytic activity in animal and human tissues and has been shown to extend lifespan in experimental models [254]. Like quercetin, fisetin modulates multiple signaling pathways associated with cancer and aging, including telomerase reverse transcriptase, oxidative stress, inflammation, cell cycle and division, apoptosis, angiogenesis, and metastasis formation [237,255,256]. Fisetin induces apoptosis selectively in senescent but not proliferating human cells and seems to be one of the best flavonoid candidates for clinical use in terms of bioavailability, efficacy, safety, feasibility, and tolerability [63,242,257]. The senotherapeutic actions of fisetin have also been recently considered for brain aging and neurodegenerative diseases, given its actions on multiple pathways related to several central nervous system (CNS) disorders, displaying a pattern of molecular targets similar to quercetin but possessing better drugability. Indeed, fisetin has been shown to prevent and mitigate age-related CNS deficits in several experimental models in vivo and is capable of reducing ROS, improving antioxidant response, exerting neuroprotection, and extending lifespan in several models of neurological disorders [258].

In summary, flavonols can be considered among the most promising (poly)phenols for further clinical research in cancer, aging, and degenerative diseases. Extensive studies have successfully highlighted some key pathways involved in their actions, and considering the overall safety registered in their use, several promising clinical trials are underway to open the way to their medical use, both in pharmacotherapy and prevention.

## Conclusion and future perspectives

Today natural (poly)phenols can be considered one of the most promising classes of pharmacologically active molecules in phytocomplexes. Although more clinical data are required, the available data on their bioavailability and their intrinsic antioxidant nature suggest that they might be endowed with a general ability to reach most biological targets and positively interfere with disease-associated processes, including systemic and tissue/organ-specific inflammatory processes. Moreover, experimental evidence has accumulated indicating their specific pharmacological activity on biological targets that extend beyond (poly)phenols’ antioxidant effect and interfere with several key pathways implicated in pivotal cellular functions, from cell death to proliferation and transformation. All of this occurs with a very low level of toxicity. Given this pleiotropic nature, it is no surprise that some of them can affect some of the most challenging pathological conditions, like the case of specific flavonols in cancer and as senolysis-mediated antiaging and anti-inflammatory strategies. Longevity, autoimmune or degenerative chronic inflammatory conditions, and cancer are probably the most meaningful examples of fields where natural (poly)phenols and their derivatives can be evaluated for their potential clinical application. Now that their pharmacology is being progressively cleared, research should focus on some aspects that can speed up their clinical application. Some of the (poly)phenols presented in this review have gone a long way along their clinical development pipeline, like quercetin and fisetin, whereas others need further clinical evidence for their application. Besides this “classical” pharmacological use in therapeutics, 3 of the most interesting and potentially groundbreaking applications of natural (poly)phenols could be represented by proactive prevention (primary or secondary) for certain cancers, longevity, and degenerative conditions (especially neuroinflammatory ones).

This approach would need a long-term dietary integration with (poly)phenol-rich phytocomplexes. The chronic application of molecules for prevention requires a personalized medicine approach, where subjects are characterized for their sensitivity and biological response potential to the polyphenol-mediated preventive strategy. The rational application of (poly)phenols in prevention would thus be a promising multifaceted strategy, where characterization of phytocomplexes would need to go along with the precise subject’s diagnostic-omics (genomics, proteomics, and/or metabolomics, as well as characterization of intestinal microbiota), in order to predetermine the potential response to the various (poly)phenols and their carriers. This approach will require further research, not just on dietary (poly)phenols but also on their key target sensitivity.

At the same time, a pharmacokinetic question remains open and needs to be answered, basically for all (poly)phenols and their phytocomplexes: can they be used in long-term preventive strategies to tackle aging or even in cancer prevention at doses compatible with their concentration in natural sources? Or should they be used as dietary integration at higher doses for short-term use? Is the interindividual gut microbiota composition a critical factor in polyphenol biotransformation and related health benefits?

Bioavailability and pharmacokinetic studies that consider the role of the gut microbiota need to be further carried out to address this issue.

## Acknowledgments

We thank group members of Solgar Italia Multinutrient S.p.A. for their thorough review and helpful discussions during the preparation of this manuscript and for their help in elaborating the search strategy.

### Author contributions

The authors’ responsibilities were as follows—VS, AB, SF, GS, AC, ID: designed the review; VS, AB, SF, SD: conducted the bibliographic research of the manuscript; VS, AB, SF, SD, GS, AC, ID: wrote the manuscript; VS, AB, SF: had primary responsibility for final content; and all authors: read and approved the final manuscript.

### Author disclosures

The authors report no conflicts of interest.

### Funding

The authors reported no funding received for this study.
